# Glutathione Suppresses Cerebral Infarct Volume and Cell Death after Ischemic Injury: Involvement of FOXO3 Inactivation and Bcl2 Expression

**DOI:** 10.1155/2015/426069

**Published:** 2015-02-04

**Authors:** Juhyun Song, Joohyun Park, Yumi Oh, Jong Eun Lee

**Affiliations:** ^1^Department of Anatomy, Yonsei University College of Medicine, Seoul 120-752, Republic of Korea; ^2^BK21 Plus Project for Medical Sciences and Brain Research Institute, Yonsei University College of Medicine, Seoul 120-752, Republic of Korea

## Abstract

Ischemic stroke interrupts the flow of blood to the brain and subsequently results in cerebral infarction and neuronal cell death, leading to severe pathophysiology. Glutathione (GSH) is an antioxidant with cellular protective functions, including reactive oxygen species (ROS) scavenging in the brain. In addition, GSH is involved in various cellular survival pathways in response to oxidative stress. In the present study, we examined whether GSH reduces cerebral infarct size after middle cerebral artery occlusion *in vivo* and the signaling mechanisms involved in the promotion of cell survival after GSH treatment under ischemia/reperfusion conditions *in vitro*. To determine whether GSH reduces the extent of cerebral infarction, cell death after ischemia, and reperfusion injury, we measured infarct size in ischemic brain tissue and the expression of claudin-5 associated with brain infarct formation. We also examined activation of the PI3K/Akt pathway, inactivation of FOXO3, and expression of Bcl2 to assess the role of GSH in promoting cell survival in response to ischemic injury. Based on our results, we suggest that GSH might improve the pathogenesis of ischemic stroke by attenuating cerebral infarction and cell death.

## 1. Introduction

Ischemic stroke resulting from obstruction of a blood vessel supplying the brain, a major cause of morbidity and mortality [1], leads to disruption of the blood-brain barrier (BBB), which subsequently causes vasogenic edema [2–4] and neuronal cell death [5]. Reperfusion after occlusion induces water entry through endothelial cells, resulting in cerebral infarction and further increases in BBB permeability [6, 7]. This reperfusion influences brain tissue by generating reactive oxygen species (ROS) [8], which contributes to oxidative stress. Glutathione (L-c-glutamyl-L-cysteinylglycine; GSH) is an intracellular thiol tripeptide present in all mammalian tissues that plays a crucial role in cellular protection against oxidant damage [9]. A decrease in cellular GSH induces the release of cytochrome c, and the redox environment thus triggers the apoptotic signaling pathway [10, 11]. In addition, a deficiency of GSH is associated with a variety of central nervous system diseases such as Alzheimer's disease [12], Parkinson's disease [13], and stroke [14, 15]. The BBB is a structural and functional barrier that regulates the exchange of many metabolites from circulating blood to brain tissue [16]. In cerebral ischemia, the BBB is disrupted, leading to brain infarction and secondary brain damage [17]. Oxidative stress following stroke results in metabolic dysfunction causing ROS accumulation [18]. GSH is reported to have a protective effect against brain vascular endothelial dysfunction, including endothelial barrier permeability [19], cell death [20], and the scavenging of ROS [21]. GSH depletion elicits endothelial oxidative stress and cell apoptosis [20, 22]. Some clinical studies demonstrate that an increased risk for stroke is associated with low levels of GSH in the brain [23, 24]. Forkhead box O (FOXO), from the O type subfamily of the forkhead transcription factor superfamily [25], regulates a series of downstream targets and is thereby involved in diverse cellular functions including the regulation of cell permeability [25–28]. Furthermore, hypoxia stress resulting from stroke triggers FOXO3-mediated dysfunction of the BBB [29]. FOXO transcriptional factors are associated with multiple mechanisms in response to oxidative stress [30], including activation of the cellular apoptotic pathway [31], several ROS-regulated mechanisms [32], and upregulation of several antioxidant enzymes such as manganese superoxide dismutase [33, 34]. FOXO3 transcription factors are downstream effectors of PI3K/Akt [35, 36]. The activity of FOXO is modulated by the phosphorylation, acetylation, and ubiquitination of PI3K/Akt [34]. FOXO phosphorylation is mainly induced through the PI3K-mediated Akt pathway, which is known to have a crucial role in cell cycle arrest and cell survival [32]. Also, FOXO3 is associated with the expression of Bcl2, which is an important antiapoptotic protein [37] in cellular protection against oxidative stress [38, 39]. In the present study, we investigated whether GSH reduces infarct size, the association of GSH with the PI3K/Akt pathway, and the influence of GSH on the expression of FOXO3 and Bcl2 after ischemic injury. Our results indicate that GSH might inhibit the effects of cerebral infarction and boost antiapoptotic signaling after ischemic stroke, suggesting that GSH may be a potent therapeutic antioxidant that can attenuate severe pathologies after ischemic stroke.

## 2. Methods and Materials

### 2.1. Animals

Male Sprague-Dawley (SD) rats (Orient, Gyeonggi-Do, Republic of Korea; 8 weeks old; 250–260 g) were subject to transient focal cerebral ischemia by intraluminal middle cerebral artery blockade with a nylon suture, as previously described [40]. After 60 min of middle cerebral artery occlusion (MCAO), blood flow was restored by withdrawing the suture, and regional cerebral blood flow was monitored using a laser Doppler flow meter (Transonic Systems, Inc., Ithaca, NY, USA). All animal procedures and experiments were performed in accordance with the Guide to the Care and Use of Laboratory Animals and were approved by the Association for Assessment and Accreditation of Laboratory Animal Care. We used at least 5 rats in all groups for study. Each measurement included 5 repeats per animal.

### 2.2. Drug Treatments

For each experiment, rats were given anesthesia (chloral hydrate, 300 mg/kg, intravenous infusion (i.v.)). GSH (Sigma-Aldrich, St. Louis, MO, USA) was dissolved in normal saline (pH 7.5). Rats were injected with GSH (500 mg/kg/mL, i.v.) 10 min after MCAO occlusion [41]. Control rats were given an equal volume of saline using the same procedure.

### 2.3. Evaluation of Infarct Volume

For evaluation of brain edema, rats were sacrificed at reperfusion 24 hr after MCAO injury. Brain slices (2-mm thick) between −22.00 mm and +4.00 mm relative to Bregma were incubated with 2% 2, 3, 5-triphenyltetrazolium chloride (TTC) (Sigma-Aldrich, St. Louis, MO, USA) at 37°C for 10 min in the dark in a drying oven and later photographed using a Nikon E 950 digital camera attached to a dissecting microscope. Infarct volume was determined from digitized images using the Quantity One software package (Bio-Rad, Philadelphia, PA, USA). Typically, 5 slices were used for analysis.

### 2.4. Cresyl Violet Staining

At reperfusion 24 hr after MCAO injury, rats were sacrificed, and brains were fixed in 3.7% formaldehyde and quickly frozen. Tissues were sectioned (20 *μ*m) coronally and sequentially dipped into xylene for 5 min, 100% alcohol for 5 min, 95% alcohol for 5 min, and 70% alcohol for 5 min. Sections were stained with cresyl violet (Sigma-Aldrich, St. Louis, MO, USA) for 3 min. After staining, slides were reacted with 70% alcohol for 5 min, 95% alcohol for 5 min, 100% alcohol for 5 min, and xylene for 5 min. Sections were then observed under a microscope equipped with a digital camera (Olympus, Tokyo, Japan).

### 2.5. Immunohistochemistry

Frozen brain sections (5 *μ*m) were cut onto clean glass slides (Thermo Scientific, Waltham, MA, USA), air-dried, and fixed in cold acetone for 10 min at −20°C. The slides were first washed in Tris-buffered saline (TBS) and then incubated with 0.3% H_2_O_2_ in methanol to quench endogenous peroxidase activity. Followed by a series of washes (three times with distilled water), the sections were blocked with 10% normal rabbit serum. Frozen brain sections (5 *μ*m) were fixed in ice-cold acetone for 20 min. To block nonspecific labeling, sections were incubated in 5% bovine serum albumin (BSA; Sigma-Aldrich, St. Louis, MO, USA) diluted in phosphate-buffered saline (PBS) for 30 min before addition of primary and secondary antibodies. Primary antibodies for claudin-5 (1 : 50; Invitrogen, Carlsbad, CA, USA) were applied to the samples for 24 hr at 4°C followed by a 90-min incubation with appropriate fluorescent secondary antibody (1 : 100; Invitrogen, Carlsbad, CA, USA) and three washes in PBS for 10 min each. After three washes in 0.1% PBS with Tween-20 (PBST), the sections were incubated with rhodamine-conjugated sheep anti-rabbit or FITC-conjugated sheep anti-mouse secondary antibody diluted to 1 : 200 with 5% BSA fraction V in 0.1% PBST for 2 hr in the dark at room temperature. After three washes in PBS, sections were incubated with 1 *μ*g/mL 4′,6-diamidino-2-phenylindole (DAPI, Sigma-Aldrich, St. Louis, MO, USA) and 2 *μ*g/mL propidium iodide (Sigma-Aldrich, St. Louis, MO, USA) for counterstaining. Tissues were then visualized under a confocal microscope (Zeiss LSM 700, Carl Zeiss, Oberkochen, Germany). The percentages of relative intensity were measured by using Image J as by referring the previous study [42].

### 2.6. Cell Culture

Murine brain endothelial cells (bEnd.3 cells; ATCC, Manassas, VA, USA) were cultured in Dulbecco's modified Eagle's medium (Hyclone Laboratories, South Logan, UT, USA) supplemented with 10% (v/v) fetal bovine serum (Hyclone Laboratories, South Logan, UT, USA) and 100 units/mL penicillin/streptomycin (Hyclone Laboratories, South Logan, UT, USA) at 37°C in a humidified atmosphere in the presence of 5% CO_2_ [43]. bEND.3 cells were used after 13 passages.

### 2.7. Hypoxia and Reperfusion (H/R) Injury

Confluent cells were transferred to an anaerobic chamber (Thermo Scientific, Pittsburgh, PA, USA) (O_2_ tension, 0.1%) and washed three times with PBS. Then, culture medium was replaced with deoxygenated, glucose-free balanced salt solution, and cells were incubated for 6 hr. Following hypoxia injury, cells were incubated for 18 hr under normal growth conditions with or without drug treatment [44].

### 2.8. Drug Treatment

GSH (Sigma-Aldrich, St. Louis, MO, USA) was dissolved in ethanol. An equivalent volume of ethanol (final: 0.01%) or water was added to control and all GSH-containing wells. bEnd.3 cells were exposed to 1 mM GSH for 3 hr before H/R injury (this concentration was chosen to study the cellular protective effect of GSH against H/R injury based on the results of our previous study). The present study consisted of four groups of cells. Normal control (NC) cells were cultured with nontreated media without H/R injury, experimental control (EC) cells were cultured in nontreated medium for 18 hr after 6 hr of H/R injury, and 1 mM GSH cells were pretreated with 1 mM GSH for 3 hr before 6 hr of H/R injury. Cells were then cultured in nontreated medium for 18 hr. Cells in the Akt inhibitor group were treated with 100 nM Akt inhibitor (Sigma-Aldrich, St. Louis, MO, USA) 3 hr before H/R injury.

### 2.9. Determination of Intracellular ROS

The level of intracellular ROS for each treatment group was measured using a fluorescent probe, 2′, 7′ -dichlorodihydrofluorescein diacetate (DCF-DA; Invitrogen, Carlsbad, CA, USA), as previously described [45]. Cells were plated at a density of 1 × 10^6^ cells/mL and treated with GSH for 24 hr. After GSH pretreatment, H/R was performed. Then, bEND.3 cells were treated with 5 *μ*M DCF-DA for 30 min at 37°C. After washing with PBS, fluorescence was measured with a microscope (Nikon TS100-F ECLIPSE) equipped with a CCD camera (Hamamatsu Photonics) [43].

### 2.10. Western Blot Analysis

After GSH pretreatment, H/R injury, and restoration, cells were washed rapidly with ice-cold PBS, scraped, and collected. Cell pellets were lysed with ice-cold RIPA buffer (Sigma-Aldrich, St. Louis, MO, USA). The lysates were centrifuged at 13,200 rpm for 1 hr at 4°C to produce whole-cell extracts. Protein content was quantified using the BCA method (Pierce, IL, USA). Protein (20 *μ*g) was separated on a 10% SDS-polyacrylamide gel and transferred onto a polyvinylidene difluoride membrane. After blocking with 5% bovine serum albumin prepared in TBS/Tween (20 nM Tris (pH 7.2), 150 mM NaCl, 0.1% Tween 20) for 1 hr at room temperature, immunoblots were incubated overnight at 4°C with primary antibodies that specifically detect phosphoinositide 3-kinase (PI3K; 1 : 2000; Cell Signaling, Danvers, MA, USA), Akt (1 : 2000; Cell Signaling, Danvers, MA, USA), phosphorylated Akt (p-Akt; 1 : 2000; Cell Signaling, Danvers, MA, USA), or *β*-actin (1 : 2000; Cell Signaling, Danvers, MA, USA). Blots were then incubated with horseradish peroxidase-linked anti-mouse or -rabbit IgG antibodies (Abcam, Cambridge, MA, USA) for 1 hr at room temperature. Enhanced chemiluminescence was performed by ECL (Pierce, IL, USA) [43].

### 2.11. RT-PCR

To examine the expression of Bcl2 in GSH-pretreated b.END3 cells under hypoxic conditions, reverse transcription-polymerase chain reaction (RT-PCR) was performed using Bcl2 primers. Briefly, samples were lysed with Trizol reagent (Invitrogen, Carlsbad, CA, USA), and total RNA was extracted according to the manufacturer's protocol. cDNA synthesis from mRNA and sample normalization was performed. PCR was performed using the following thermal cycling conditions: 10 min at 95°C; 40 cycles of denaturing at 95°C for 15 sec, annealing at 65°C for 30 sec, and elongation at 72°C for 30 sec; final extension at 72°C for 10 min; and holding at 4°C. PCR was performed using the following primers (5′ to 3′): Bcl2 (F): AAGCTGTCACAGAGGGGCTA, (R): CAGGCTGGAAGGAGAAGATG; GAPDH (F): GGCATGGACTGTGGTCATGAG, (R): TGCACCACCAACTGCTTAGC. PCR products were electrophoresed in 1.5% agarose gels and stained with ethidium bromide.

### 2.12. Statistical Analysis

Statistical analyses were carried out using SPSS 18.0 software (IBM Corp., Armonk, NY, USA). Data are expressed as the mean ± standard error of the mean of three independent experiments. The statistical significance of group differences was determined by one-way analysis of variance (ANOVA) followed by Bonferroni* post hoc *multiple comparison tests. The statistical significance of differences from the MCAO group (i.e., EC group) was determined by* t*-tests. Differences were considered statistically significant at *P* < 0.05. (^*^ *P* < 0.05, ^**^ *P* < 0.001)

## 3. Results

### 3.1. GSH Reduced Infarct Volume following Cerebral Ischemia

**Figure 1:**
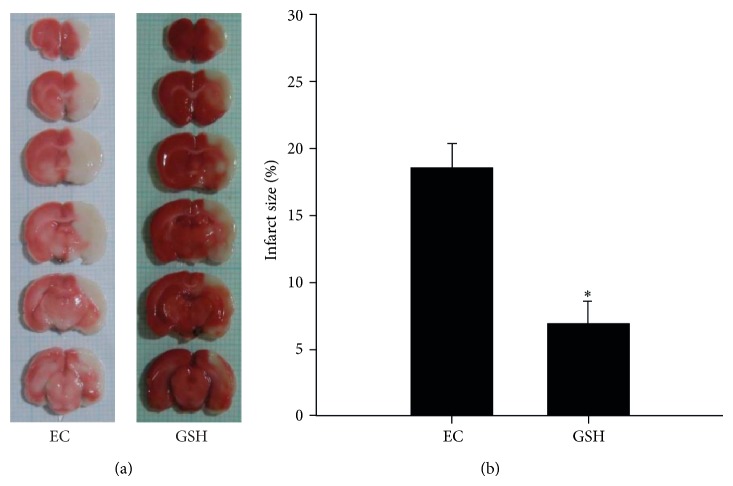
Measurement of infarct volume in MCAO rat brain at reperfusion 24 hr after MCAO injury. (a) At reperfusion 24 hr after MCAO injury, TTC staining showed that white areas were damaged by ischemic injury. White areas reduced more than 2-fold changes in the GSH treatment group compared to experimental control (EC) group. (b) Brain edema (%) was measured at reperfusion 24 hr after MCAO injury. The quantitative graph shows that brain edema was significantly reduced in GSH treatment group compared with EC group. Each experiment included 5 repeats per animal. Data were expressed as mean ± S.E.M. Statistical significance with EC group was determined by *t*-test. Differences were considered significant at ^*^ *P* < 0.05. EC: experimental control; reperfusion 24 hr after MCAO injury group, GSH: GSH treatment and reperfusion 24 hr after MCAO injury group.

To investigate whether GSH affects vascular permeability in the animal brain, we measured infarct volume at reperfusion 24 hr after MCAO injury using TTC staining (Figure 1(a)). White areas indicate damaged brain areas due to ischemia (Figure 1(a)). We sacrificed 6 rats in all groups. The graph shows the percentage of injured ipsilateral hemisphere relative to uninjured ipsilateral hemisphere for the MCAO and GSH groups (Figure 1(b)). The percentage of infarcted area in the MCAO group was >17%, whereas the percentage of brain edema after GSH treatment was <8%. Infarct volume (%) was significantly reduced in the GSH group compared with the MCAO group. This graph included the observation results of 5 repeats per animal. Our findings indicate that GSH treatment reduced cerebral infarct volume after ischemic brain injury.

### 3.2. Assessment of GSH-Induced Morphological Alterations Using Cresyl Violet Staining

**Figure 2:**
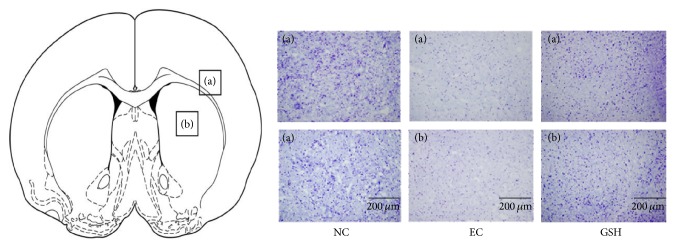
The histological assessment using cresyl violet staining after ischemic injury. Atlas of rat brain mainly presents the corpus callosum, cerebral cortex (a), and striatum (b). Cresyl violet staining indicated that considerable cell loss was observed in the 24 hr MCAO group whereas more healthy cell bodies in striatum and cortex were observed in MCAO with GSH treatment group. Each assessment included 5 repeats per animal. (a) Cortex, (b) striatum, scale bar: 200 *μ*m, NC: normal control group, EC: experimental control, reperfusion 24 hr after MCAO injury group, and GSH: GSH treatment and reperfusion 24 hr after MCAO injury group.

Cresyl violet staining was performed at reperfusion 24 hr after MCAO injury to assess the extent of ischemia-induced damage in the striatum and cortex (Figure 2). In the NC group (without MCAO injury or GSH treatment), intact cellular structure was observed in both the cortex and striatum. In the MCAO group (i.e., EC group), small, shrunken cell bodies and damaged tissue was observed in the ischemic cortex and striatum (Figure 2). In the GSH-treated group (GSH treatment and MCAO injury), damaged cells were fewer in number compared with the EC group, and we observed healthy round cells in the ischemic cortex and striatum (Figure 2). This result included 5 repeats observation result per animal.

### 3.3. GSH Prevents BBB Disruption following Cerebral Ischemia

**Figure 3:**
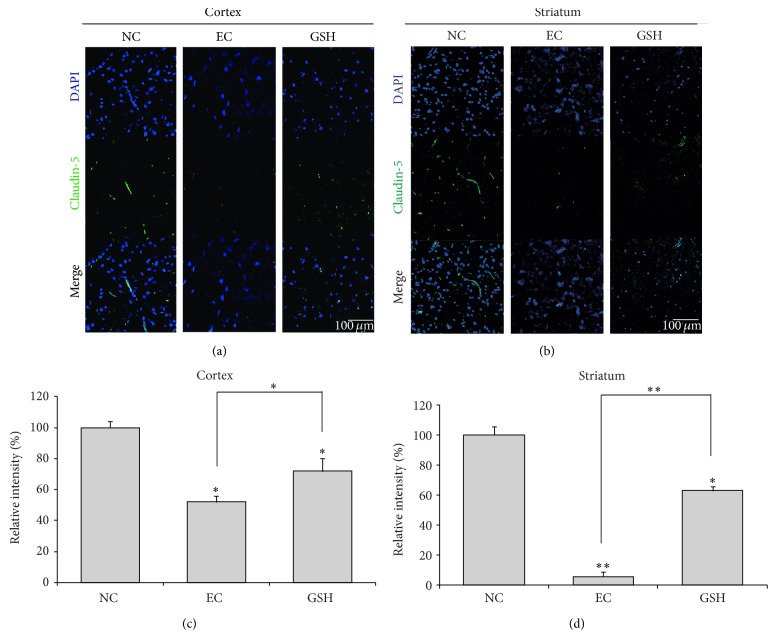
Immunochemical image for confirmation of preserved claudin-5 expression by GSH treatment. (a) Immunochemical images showed that claudin-5-positive cells (green) were particularly reduced in rat cortex of experimental control (EC) group. In GSH treatment group, claudin-5 protein expression was observed in rat cortex compared to the EC group. (b) Ischemic striatum (EC group) showed reduced claudin-5 expression while in GSH treatment group, claudin-5-positive cells were retained in rat striatum owing to GSH treatment. (c) The graph showed the relative intensity percentage (%) of claudin-5 expression in the cortex region to compare the normal control group (as considering that the normal control group's intensity is 100%). (d) The graph showed the relative intensity percentage (%) of claudin-5 expression in the striatum region to compare the normal control group. Each experiment included 5 repeats per animal. The statistical significance of group differences was determined by one-way analysis of variance (ANOVA) followed by Bonferroni* post hoc *multiple comparison tests. Differences were considered significant at ^*^ *P* < 0.05, ^**^ *P* < 0.001. Scale bar = 100 *μ*m, claudin-5: green, 4′, 6-diamidino-2-phenylindole (DAPI): blue. NC: normal control group, EC: experimental control, reperfusion 24 hr after MCAO injury group, and GSH: GSH treatment and reperfusion 24 hr after MCAO injury group.

We conducted immunohistochemistry using claudin-5 antibody at reperfusion 24 hr after MCAO injury to examine changes in the expression of this BBB junction protein in the cortex (Figure 3(a)) and striatum (Figure 3(b)). In the NC group, claudin-5 was considerably expressed in both the cortex (Figure 3(a)) and striatum (Figure 3(b)). However, claudin-5 expression was attenuated in both the cortex (Figure 3(a)) and striatum (Figure 3(b)) at reperfusion 24 hr after MCAO injury (EC group). In the GSH-treated group, claudin-5 expression was increased in both the cortex (Figure 3(a)) and striatum (Figure 3(b)) compared with the EC group. Figures 3(c) and 3(d) graphs expressed the relative intensity compared to the normal control group (Figures 3(c) and 3(d)). The relative intensity was measured by using Image J by following the previous study [42] to show to the differences of claudin-5 expression between groups in spite of indirect quantification method. These results included 5 repeats per animal. Based on these results, we suggest that GSH may inhibit the degradation of claudin-5 in the ischemic brain and protect against BBB disruption.

### 3.4. GSH Decreases H/R-Induced ROS Production

**Figure 4:**
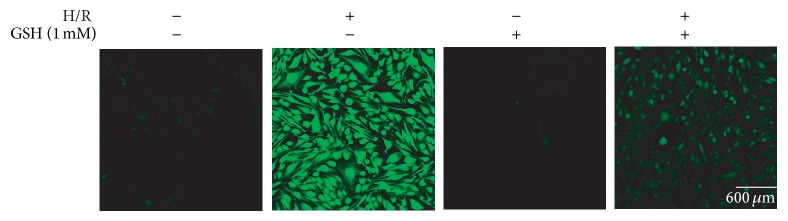
Immunocytochemistry to measure ROS generation in bEND.3 cells after H/R-induced injury. bEND.3 cells were treated with GSH for 3 hr before H/R injury. ROS levels were measured using DCF-DA. ROS levels in GSH treatment group were almost the same with the NC (normal control) group. ROS levels in bEND.3 cells were increased in H/R injury exposed group. Under H/R injury, ROS levels in the GSH pretreatment group were decreased compared to H/R injury exposed group. GSH attenuated the H/R-induced increase in DCF-DA-positive cells (green). Each experiment included 4 repeats per condition. 2′, 7′-Dichlorodihydrofluorescein diacetate (DCF-DA): green, scale bar = 600 *μ*m. H/R: hypoxia 6 hr and reperfusion 18 hr injury group.

We measured ROS levels using DCF-DA reagent, a fluorescent dye that visualizes ROS, in b.END3 cells. DCF-DA-positive cells increased in number after H/R injury. ROS levels in the 1 mM GSH pretreatment group were not largely different from those in the NC group. In the H/R injury group, ROS levels were increased compared with those in the NC group. This increase was partially blocked by pretreatment with 1 mM GSH (Figure 4). The H/R injury group with 1 mM GSH pretreatment showed a clear decrease in the number of DCF-DA-positive cells compared with the H/R injury group. This result suggests that GSH inhibits H/R-induced ROS production in b.END3 cells.

### 3.5. GSH Attenuates the Death of b.END3 Cells after H/R Injury via PI3K/Akt Activation and FOXO3 Inactivation

**Figure 5:**
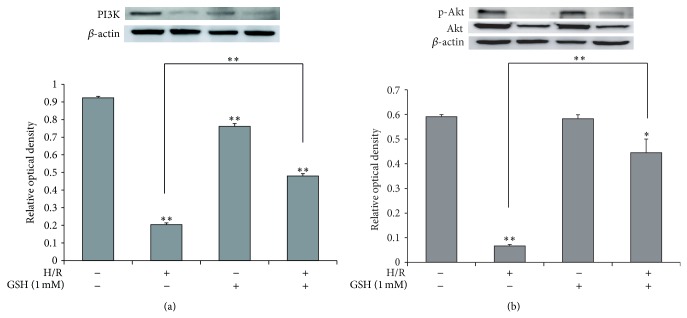
The measurement of PI3K, Akt expression in brain enthothelial cells after H/R-induced injury. (a) Western blotting showed that the relative protein level of PI3K was reduced in EC compared to the NC group. The protein level of PI3K was increased in 1 mM GSH groups, compared to the EC group. The bar graph shows the quantification of PI3K protein in all groups. (b) Western blotting showed that the relative protein level of phosphor-Akt was reduced in EC compared to the NC group. The protein level of phosphor-Akt was increased in 1 mM GSH groups, compared to the EC group. The bar graph shows the quantification of phosphor-Akt/Akt protein in all groups. *β*-actin was used as an internal control. Each experiment included 3 repeats per condition. Data are expressed as mean ± S.E.M. (^*^ *P* < 0.05, ^**^ *P* < 0.001). Phosphoinositide 3-kinase (PI3K), protein kinase B (Akt), and phosphorylated Akt (p-Akt).

**Figure 6:**
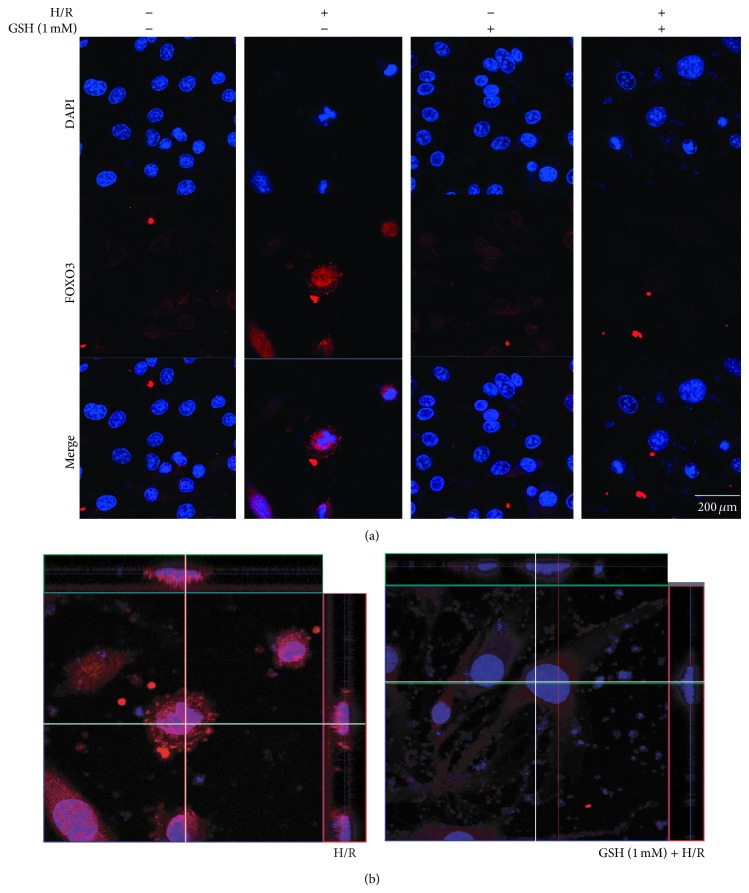
The immunocytochemical image to evaluate the activation of FOXO3 in H/R injured bEnd.3 cells. (a) The activation of FOXO3 was evaluated with immunocytochemistry. This image showed the translocation of FOXO3 to the nucleus of b.END3 cells in the H/R group was increased compared with the normal control group. 1 mM GSH pretreatment group with H/R injury group showed suppression of FOXO3 translocation to the nucleus compared with the hypoxia group. 1 mM GSH decreased the activation of FOXO3 under hypoxic condition. (b) This ortho image shows the activation of FOXO3 by suggesting the translocation of FOXO3 into the nucleus. The translocation of FOXO3 into the nucleus was evidently showed in the H/R group whereas the translocation of FOXO3 into the nucleus was reduced by treating GSH in spite of the H/R stress. Each experiment included 3 repeats per condition. Scale bar = 400 *μ*m, H/R: hypoxia 6 hr, and reperfusion 18 hr injury group, GSH + H/R: GSH pretreatment and hypoxia 6 hr and reperfusion 18 hr injury group.

To investigate whether PI3K/Akt signaling is activated in H/R-induced stress, we first measured the expression of PI3K by western blot analysis (Figure 5(a)), as PI3K expression correlates with the activation of cell survival signaling. H/R-injured cells showed an obvious suppression of PI3K expression, whereas PI3K activation in H/R-injured cells with 1 mM GSH pretreatment was not largely different from that in the EC group (Figure 5(a)). Next, we examined the phosphorylation of Akt by western blot analysis (Figure 5(b)). Phosphorylation of Akt is associated with activation of Akt signaling and cell survival. Our results suggest that the expression of phosphorylated Akt protein in the EC group is attenuated compared with that in the NC group. The relative portion of phosphorylated Akt in the 1 mM GSH group was similar to that in the EC group. However, expression of phosphorylated Akt in the 1 mM GSH treatment group was higher than that in the EC group (Figure 5(b)). In addition, more translocation of FOXO3 occurred in the GSH-treated group than in the EC group despite the same H/R injury (Figures 6(a) and 6(b)). These results show that GSH may activate PI3K/Akt signaling and inhibit the activation of FOXO3 in H/R-injured brain endothelial cells.

### 3.6. GSH Promotes the Expression of Bcl2 after H/R Injury

**Figure 7:**
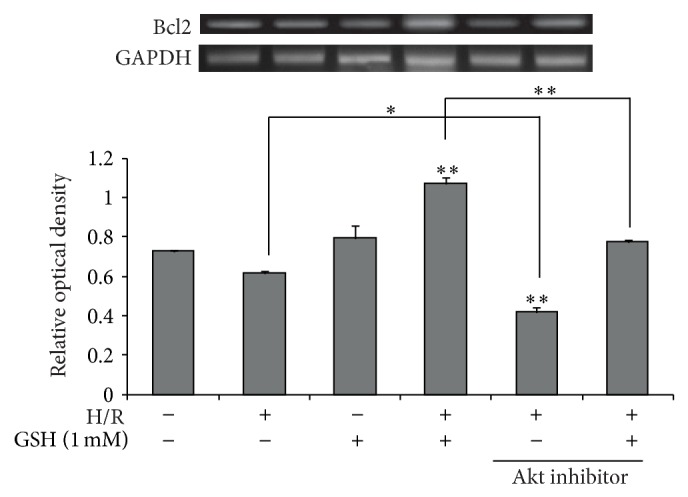
The mRNA level of Bcl2 in hypoxia exposed b.End3 cells. The mRNA level of Bcl2 was measured using RT-PCR. The mRNA level of Bcl2 was decreased in the hypoxia injury group compared to the normal control group. 1 mM GSH increased the mRNA level of Bcl2 in hypoxia injured b.END3 cells. The mRNA level of Bcl2 was increased in the hypoxia injury group compared to the normal control group. 1 mM GSH reduced mRNA level of Bcl2 in hypoxia-injured b.END3 cells. In Akt inhibitor groups, the mRNA level of Bcl2 shows the reduction compared to the no inhibitor treatment group, respectively. The bar graph shows the quantification of Bcl2 mRNA in all groups. GAPDH was used as an internal control. Each experiment included 5 repeats per condition. Data are expressed as mean ± S.E.M. (^*^ *P* < 0.05, ^**^ *P* < 0.001).

To examine whether PI3K/Akt signaling contributes to the expression of Bcl2 in H/R injury, we measured the mRNA level of Bcl2 by RT-PCR (Figure 7), as Bcl2 correlates with activation of cell survival signaling. H/R-injured cells showed an obvious suppression of Bcl2 expression, whereas Bcl2 activation in H/R-injured cells with 1 mM GSH pretreatment was markedly higher than that in the EC group (Figure 7). Under the situation that the activation of Akt was inhibited by an Akt inhibitor, Bcl2 expression was further reduced by GSH pretreatment in H/R-injured cells (Figure 7). These results show that GSH may activate PI3K/Akt signaling and thereby promote the expression of Bcl2 as a modulator of cell survival in H/R-injured b.END3 cells.

## 4. Discussion

Ischemic stroke causes brain damage and alterations in GSH redox status [46, 47]. Cerebral infarction volume following ischemic stroke is increased in GSH-deficient mice [48, 49]. In the present* in vivo* study, we demonstrated that GSH reduces brain infarct volume after MCAO injury in the rat brain. Junction proteins such as cadherins, *α*-catenin, occluding, claudin, and *β*-catenin are components of the BBB [50]. When the brain is exposed to stress, the first barrier is brain endothelial cells sealed by tight junctions [51], which form a junction complex between endothelial cells [52, 53]. The endothelial cells connected by junction proteins play a critical role in maintaining the integrity of the BBB to protect the brain [54], and tight proteins regulate paracellular permeability [55]. The results of the present study suggest that GSH preserves the loss of the tight junction protein claudin-5 after MCAO injury. Moreover, to determine the specific mechanism underlying the cellular protection provided by GSH, we investigated the cellular mechanism through* in vitro* experiments. In ischemic stress, the increased production of ROS in the brain triggers signaling cascades leading to inflammation, apoptosis, and ultimately memory and motor deficits [56]. The appropriate regulation of ROS by cellular antioxidant redox enzymes such as GSH is essential to prevent oxidative stress-induced brain damage [57]. In the present* in vitro* experiments, we confirmed that GSH attenuates the production of ROS in brain endothelial cells after ischemic injury. Hypoxia induces FOXO3-mediated dysfunction of the BBB [29]. Therefore, we investigated the activation of FOXO3, which is associated with disruption of the BBB [29] and increased proapoptotic gene expression [58], as well as the activation of PI3K/Akt signaling, which is an upstream target of FOXO3 transcription [58, 59] and a cell survival pathway [60, 61]. FOXO3 modulates diverse cellular and physiological mechanisms including metabolism, apoptosis, and cellular longevity [34, 62–64]. The H_2_O_2_-induced decrease in cell viability is mediated by a FOXO3-dependent mechanism [65]. Deacetylation of FOXO3 protects mitochondria against oxidative stress by boosting mitochondrial homoeostasis [66] and reduces the production of cellular ROS [67, 68]. FOXO3 mediates ROS-induced apoptosis through the expression of ROS scavenging enzymes [69]. In the present study, we found decreased activation of FOXO3 translocation into the nucleus in the GSH-treated MCAO injury group. This reduction may be related to a decreased production of ROS by GSH treatment. In addition, one study demonstrated that FOXO3 increases hypoxia-induced BBB hyperpermeability [29]. Recent studies show that hypoxic conditions promote the translocation of FOXO3 into the nucleus of endothelial cells [29, 70] and increases FOXO3, triggering hypoxia-induced degradation of junction proteins such as VE-cadherin and claudin-5 [29]. Based on our results, GSH may prevent the degradation of the tight junction protein claudin-5 following ischemic stroke, which may involve the reduced translocation of FOXO3 into the nucleus. Moreover, phosphorylation of FOXO by Akt leads to the holding of FOXO in the cytoplasm and the blockade of target gene transcription [35]. Akt promotes cell survival by regulating FOXO transcription factors [71]. Activation of FOXO3 also induces apoptosis and cell cycle arrest in endothelial cells by suppressing heat shock protein 70 expression [72, 73]. Activation of FOXO3 causes an increased expression of the antiapoptotic protein Bcl2 [37]. In addition, overexpression of Bcl2 leads to an increase in the cellular content of GSH [74, 75]. By contrast, Bcl2 deficient-mice show reduced GSH levels in brain tissue and exhibit an increased risk of neuronal cell death [76]. Considering the results of the present study, we assume that an increased expression of Bcl2 may protect brain tissue from damage due to ischemic stroke. Briefly, we suggest three possibilities: (1) GSH attenuates cerebral infarct volume after ischemic stroke, (2) GSH preserves the disruption of BBB after ischemic injury, and (3) GSH improves the survival of brain endothelial cells by promoting the PI3K/Akt pathway, inhibiting the translocation of FOXO3 into the nucleus, and boosting the expression of Bcl2. Thus, we thought that this study would provide the basic data to understand the role of GSH in ischemic stroke. Ultimately, we suggest the need for further study regarding the role of GSH in alleviating severe brain pathologies caused by ischemic stroke.
